# IDH1 mutation produces R-2-hydroxyglutarate (R-2HG) and induces mir-182-5p expression to regulate cell cycle and tumor formation in glioma

**DOI:** 10.1186/s40659-024-00512-2

**Published:** 2024-05-17

**Authors:** Haiting Zhao, Li Meng, Peng Du, Xinbin Liao, Xin Mo, Mengqi Gong, Jiaxin Chen, Yiwei Liao

**Affiliations:** 1 National Clinical Research Center for Geriatric Disorders, Xiangya Hospital, Central South University, Changsha, 410008 P.R. China; 2 Department of Neurology, Xiangya Hospital, The Central South University (CSU), Changsha, 410008 P.R. China; 3 Department of Neurosurgery, Xiangya Hospital, Central South University (CSU), Changsha, 410008 P.R. China; 4 Department of Radiology, Xiangya Hospital, Central South University (CSU), Changsha, 410008 P.R. China; 5 Department of Neurosurgery, The Second Affiliated Hospital, Xinjiang Medical University, Urumqi, 830063 PR China

**Keywords:** Gliomas, *IDH1* mutation, R-2HG, miR-182-5p, Cell cycle, CS-NPs(antagomir-182-5p)

## Abstract

**Background:**

Mutations in isocitrate dehydrogenase 1 and 2 (*IDH1* and *IDH2*), are present in most gliomas. *IDH1* mutation is an important prognostic marker in glioma. However, its regulatory mechanism in glioma remains incompletely understood.

**Results:**

miR-182-5p expression was increased within *IDH1*-mutant glioma specimens according to TCGA, CGGA, and online dataset GSE119740, as well as collected clinical samples. (R)-2-hydroxyglutarate ((R)-2HG) treatment up-regulated the expression of miR-182-5p, enhanced glioma cell proliferation, and suppressed apoptosis; miR-182-5p inhibition partially eliminated the oncogenic effects of R-2HG upon glioma cells. By direct binding to Cyclin Dependent Kinase Inhibitor 2 C (*CDKN2C*) 3’UTR, miR-182-5p inhibited *CDKN2C* expression. Regarding cellular functions, *CDKN2C* knockdown promoted R-2HG-treated glioma cell viability, suppressed apoptosis, and relieved cell cycle arrest. Furthermore, *CDKN2C* knockdown partially attenuated the effects of miR-182-5p inhibition on cell phenotypes. Moreover, *CDKN2C* knockdown exerted opposite effects on cell cycle check point and apoptosis markers to those of miR-182-5p inhibition; also, *CDKN2C* knockdown partially attenuated the functions of miR-182-5p inhibition in cell cycle check point and apoptosis markers. The engineered CS-NPs (antagomir-182-5p) effectively encapsulated and delivered antagomir-182-5p, enhancing anti-tumor efficacy in vivo, indicating the therapeutic potential of CS-NPs(antagomir-182-5p) in targeting the miR-182-5p/*CDKN2C* axis against R-2HG-driven oncogenesis in mice models.

**Conclusions:**

These insights highlight the potential of CS-NPs(antagomir-182-5p) to target the miR-182-5p/*CDKN2C* axis, offering a promising therapeutic avenue against R-2HG’s oncogenic influence to glioma.

**Supplementary Information:**

The online version contains supplementary material available at 10.1186/s40659-024-00512-2.

## Introduction

Mutations in tricarboxylic acid cycle (TCA cycle) enzymes, such as isocitrate dehydrogenase 1 and 2 (*IDH1* and *IDH2*), are present in several cancers, particularly gliomas [1, 2]. Heterozygous mutations in *IDH1* and *IDH2*, especially those in *IDH1* have been discovered within 70–80% of cases of WHO grade II and III astrocytoma, oligodendrogliomas, and secondary glioblastomas [3–5]. Nowadays, mutated *IDH1* even defines a different molecular subtype of diffuse glioma [1, 6]. Therefore, investigating the role and mechanism of *IDH1* mutation in glioma pathogenesis could pave the way for the development of targeted therapeutic strategies that specifically address the effects of IDH1 mutations in gliomas.

The effects of *IDH1* mutation upon gliomas are complex and seemingly paradoxical. Glioblastoma patients carrying an *IDH1* mutation show better prognosis; however, the median overall survival remains about 31 months for gliomas patients [1] and 44 months for glioblastoma multiforme (GBM) patients [6]. Notably, a mutation in *IDH1* has been found to be an early event during the onset of gliomas [7, 8] and thus may exert a vital effect on the initiation of disease. This early onset of *IDH1* mutations suggests a more complex role than a mere loss of the enzyme’s normal function. Rather than simply losing its enzymatic activity, the *IDH1* mutation imparts the enzyme with a neomorphic activity, leading to the reduction of α-ketoglutarate to the oncometabolite R(−)-2-hydroxyglutarate (R-2HG). Reportedly, accumulated 2HG in the brain might increase the risk of developing brain tumors [9–11]. *IDH1* mutation in vivo promote the growth of gliomas and several other malignancies through elevating stem cell number and affecting differentiation [12–16]. *IDH1*^*R132H*^ (mutations occur at a single amino acid residue of IDH1, arginine 132 mutated to histidine) is the most common IDH mutation, present in ∼ 90% of IDH-mutant cases [17]. For instance, a high-throughput screen was employed to identify AGI-5198 and MRK-A, selective inhibitors that target *IDH1*^*R132H*^, which have been discovered to inhibit the generation of the oncometabolite R-2HG and the development of *IDH1*^*R132H*^-overexpressing gliomas dose-dependently [18, 19]. Given the association of *IDH1* mutations with both an improved prognosis and a pivotal role in disease onset, selective inhibitors targeting *IDH1* or R-2HG related signaling pathways are attractive strategies for gliomas treatment regimens [20].

microRNAs (miRNAs) are noncoding oligonucleotides capable of regulating messenger RNA (mRNA) transcript translation and levels in the cytoplasm. One miRNA can regulate several genes as its targets, so miRNA expression profiling may more precisely stratify biologically and clinically relevant subgroups than standard mRNA expression profiling [21, 22]. Furthermore, the ability to modulate or simulate these oligonucleotides therapeutically via synthetic techniques suggests that these studies may have clinical value [22]. Unlike mRNAs, miRNAs are biologically stable and are not susceptible to quick degradation by RNases, facilitating their potentials as prognostic and diagnostic biomarkers, as well as therapeutic targets for gliomas [23–25]. Numerous miRNAs have been repeatedly found to be dysregulated in many investigations of miRNA expression in gliomas, such as those conducted as part of The Cancer Genome Atlas (TCGA; https://www.cancer.gov/about-nci/organization/ccg/research/structural-genomics/tcga) and Chinese Glioma Genome Atlas (CGGA; http://www.cgga.org.cn/) projects. miRNAs that are dysregulated during glioma development, aiding or hindering tumorigenesis.

While direct miRNA delivery to the brain has shown promise, a less invasive approach is preferable due to inherent challenges such as reduced resistance to RNase degradation, limited stability, and suboptimal cell uptake of miRNAs [26]. Nanoparticles (NPs), especially those fabricated from natural polymers like chitosan (CS), present a promising avenue. CS, a biodegradable and biocompatible polysaccharide consisting of D-glucosamine and N-acetyl-D-glucosamine units connected with b-(1,4) glycosidic linkages [27, 28]. Its inherent polycationic nature facilitates binding to negatively-charged therapeutics, including miRNAs, ensuring their stability, protection against degradation, and enhanced cellular uptake [27]. Given the constraints in miRNA application due to their instability and delivery challenges, the progression of an effective delivery system like CS-NPs shows to be pivotal [26]. Chitosan has great potential in the delivery of polynucleotides because of its excellent biological qualities: it is biocompatible, biodegradable, mucoadhesive and non-toxic, bridging the gap between the therapeutic potential of miRNAs and their practical application [29, 30].

In this study, differentially expressed miRNAs were analyzed using miRNA expression profiles in *IDH1*-mutant and *IDH1*-wildtype glioma based on TCGA, CGGA, and online dataset GSE119740; miR-182-5p was selected. IDH1 mutant glioma cells were subjected to treatment with R-2HG and examined for the expression of miR-182-5p and its functions upon R-2HG-treated glioma cells. We analyzed downstream targets of miR-182-5p, investigated the predicted binding of miR-182-5p to target, miR-182-5p regulation of target, and the dynamic effects of the miR-182-5p/target axis on R-2HG-treated glioma cells and xenograft tumor models. CS-NPs(antagomir-182-5p) was synthesized, the characteristics were confirmed, and the anti-tumor effects were investigated in xenograft tumor models in mice.

## Materials and methods

### Clinical sampling

A total of 12 glioma tissue samples (6 with *IDH1* mutation and 6 with wild-type *IDH1*) were obtained from patients receiving surgery at Xiangya Hospital. The glioma *IDHl* mutation was assessed by postoperative pathological diagnosis of immunohistochemistry and DNA sequencing (Cheerland, Shenzhen, China). All patients involved in the present study did not receive any preoperative radiotherapy or chemotherapy. The pathological stage, grade, and nodal condition were all examined by an experienced pathologist. All experiments were conducted under the approval of the Research Ethics Committee of the Xiangya Hospital. Each patient was consented in a written informed consent form. All the tissues were fixed in formalin or kept at -80 °C before further experiments.

### Bioinformatics analysis

The microRNA expression data of CGGA (http://www.cgga.org.cn/), TCGA-GBMLGG, and GEO public database (GSE119740) were analyzed to screen differentially expressed miRNAs (DEMs) between *IDH1* mutant- and wild-type glioma samples. “Limma” package (version 3.58.1) and “pheatmap” package (version 1.0.12) from R software (version 4.0.0) was applied to analyze and exhibit the DEMs and miRNAs exhibiting fold changes ≥ 0.4 and *P*-values ≤ 0.05 were chosen as significantly DEMs.

### RNA extraction and quantitative real-time PCR (qRT-PCR)

TRIZOL™ (Invitrogen, Carlsbad, USA) was employed to extract total RNA from cells or tissue samples, PrimeScript® Stra Strand Synthesis Kit (TaKaRa, Tokyo, Japan) was utilized to reverse transcribe 2.0 µg of total RNA for synthesis. The quantitative PCR was performed using QuantiTect® SYBR® Green RT-PCR Kit (QIAGEN, Dusseldorf, Germany). mature miR-182-5p, pre-miR-182-5p and CDKN2C expression levels were quantified using the 2^−ΔΔCt^ method and normalized using U6 or GAPDH as an internal reference. The primer sequences used in qRT-PCR assay are listed in Table S1.

### Cell lineage and cell culture

The human brain glioma cell lines BT142 (ASC-1018) with an endogenous R132H mutation in *IDH1* [31] were purchased from ATCC, USA. U251-MG were purchased from Procell (Wuhan, China). NeuroCult NS-A Proliferation kit (StemCell Technologies, Vancouver, Canada) was employed to culture BT142 cells. U251-MG cells were cultured in Dulbecco’s modified eagle media (DMEM, Gibco, Waltham, USA) supplemented with 10% of FBS (Gibco). U251-MG^*R132H*^ cells were obtained via lentivirus transduction of the pLVX-Puro vector (Oribio, Changsha, China) carrying *IDH1 R132H* variants into U251-MG cells. Stable expression cell lines were selected via addition of puromycin (1–2 µg/mL, MedChem Express, Monmouth Junction, USA), side by side with control cells [32, 33]. These two cells were cultured at 37℃ in a 5% CO_2_ atmosphere. For R-2HG treatment, the glioma cells were espoused to 100 µM R-2HG for 24 h.

### Cell viability by CCK-8

A CCK-8 kit (Sigma-Aldrich, Saint Louis, USA) was applied to assess the viability of target cells. The transfected cells (1 × 10^4^ cells/well) were planted on 96-well plates and cultured for 48 h. After that, CCK-8 solution (10 μl/well) was supplemented, and then incubated for 4 h. Using a microplate reader, the absorbance value was obtained at 450 nm.

### Cell apoptosis by Flow cytometry

Trypsin digestion (without EDTA) was employed to collect cells. Cell apoptosis was detected using an Annexin V-fluorescein isothiocyanate (FITC)/propidium iodide (PI) Apoptosis Detection Kit (Vazyme, Nanjing, China) as directed by the protocol of the manufacturer and previous research [34]. The data were analyzed using flow cytometry (Novocyte, Agilent, Santa Clara, USA).

### Cell transfection

GenePharma (Shanghai, China) was applied to synthesize the antagomir-182-5p, agomir-182-5p, small interfering RNA targeting *CDKN2C* (si-*CDKN2C*) and corresponding scramble controls, which were then transfected to target cells using Lipofectamine 3000 (Invitrogen). The sequences of antagomir-182-5p, agomir-182-5p, and si-*CDKN2C* are listed in Table S1.

### Immunoblotting

The RIPA buffer was used to extract the total protein (Solarbio, Beijing, China). Protein quantification was done with the BCA Protein Quantification Kit (Vazyme). After that, following electrophoresis by SDS-PAGE, the separated proteins (50 µg) were electroblotted from the gel on PVDF membranes (Sigma-Aldrich). followed by an overnight treatment at 4 °C with primary antibodies: CDKN2C (ab192239, Abcam, Cambridge, USA), Cyclin D1 (60186-1-Ig, Proteintech, Wuhan, China), RB1 (ab181616, Abcam), p-RB1 (ab184796, Abcam), CDK4 (11026-1-AP, Proteintech), Bcl-2 (12789-1-AP, Proteintech), BAX (50599-2-Ig, Proteintech), and then 1.5-h interaction with secondary antibody (Abcam) at room temperature (RT). Chemiluminescence was seen with the ECL kit (Vazyme).

### Dual-luciferase reporter assay

The sequences of *CDKN2C* 3’UTR was amplified and cloned into the psiCheck-2 plasmid (Promega, Madison, USA), which included the wild- or mutant-type binding sites of miR-182-5p. Then, using Lipofectamine 3000, the generated vectors (*CDKN2C* 3’UTR-wt (wild-type) and *CDKN2C* 3’UTR-mut (mutant-type)) were transfected into target cells together with antagomir-182-5p or agomir-182-5p. Lastly, the Dual-Luciferase Reporter Assay Kit (Promega) and multiple function microplate reader (Bio-rad, Hercules, USA) were employed to measure the luciferase activity.

### Xenograft mouse models

BALB/c nude mice (4 weeks old) were procured from Hunan SLAC laboratory animal company (Changsha, China) for in vivo research. The experiments were carried out in complete accordance with the procedures approved by the Ethics Committee of Xiangya Hospital of Central South University. For subcutaneous xenografts, each nude mouse’s right flank was given subcutaneous injection with 5 × 10^6^ U251-MG cells (in a total volume of 0.1 ml cell suspension). After tumor formation, the nude mice were then randomized into eight groups (six mice for each). As for in vivo function miR-182-5p and R-2HG, every 7 days, antagomir (5 nmol in 20μl) and/or R-2HG (0.5 µM in 20 µl) was administered via intratumoral injection for 4 weeks. As for the function of antagomir-loaded CS-NPs, either antagomir (5 nmol in 20 µl) was administered via intratumoral injection or an equivalent amount of antagomir-loaded CS-NPs was given, and this regimen was continued for a duration of 4 weeks. Tumor volumes were assessed and computed using the following formula: volume (mm^3^) = length×width^2^/2. After sacrificing the mice, the xenograft tumor cells were separated at the tumor endpoints. Tumor weight was determined. H&E staining was performed to evaluate the pathological alterations in tumor tissue samples. qRT-PCR was conducted to determine miR-182-5p levels within tumor tissue samples.

### Hematoxylin and eosin staining (H&E staining)

Mice were anesthetized and sacrificed at the end of the experiment, and tumors were extracted from mice. Tumors were fixed with 4% paraformaldehyde, dehydrated, and embedded with paraffin. Paraffin-embedded blocks were sectioned into 4-µm-thick serial slices, deparaffinized using xylene, and rehydrated using decreasing concentrations of alcohol (100%, 95% and 70%). Slices were washed in dH_2_O, subjected with staining with H&E solution in sequence, and then rinsed in dH_2_O. Next, slices were dehydrated using decreasing concentrations of alcohol and subjected to immersion in xylene prior to mounting in Permount.

### Preparation of CS-NPs

CS-NPs were synthesized using complex coacervation method as described by Mao [21]. Herein, chitosan (448,869, Sigma-Aldrich) with the following properties was utilized: molecular weight: 50,000-190,000 Da (based on viscosity: 20–300 cP). In short, chitosan was added into 1% acetic acid (0.5 mg/ml), which were stirred at RT for 20–24 h (hour) using a magnetic stirrer, and pH of the solution was adjusted to 6 using NaOH solution. Then, antagomir-182-5p were added into chitosan solution to 50 µg/ml. The chitosan and antagomir-182-5p mixture were stirred for 1 h at RT and ultrasonic for 30 min. The Sodium Tripolyphosphate (TTP) solution (50 mg/ml) was added dropwise to the chitosan and antagomir-182-5p mixture at N/P ratio of 50 under stirring for 1 h at RT and ultrasonic for 1 h. Lastly, 30-min centrifugation (15,000 rpm/min) was carried out to collect CS-NPs.

### Physicochemical characterization of CS-NPs(antagomir-182-5p)

A Zetasizer Nano ZS (Malvern Instruments, Malvern, UK) was employed to assess the size and zeta potential of the prepared CS-NPs (antagomir-182-5p). The assessments were carried out three times at pH 7.4 at 25℃. Transmission Electron Microscopy (TEM) was utilized to perform the morphological examination of CS-NPs (antagomir-182-5p). Next, CS-NPs solution was dropped onto a carbon-coated copper grid and dried at RT. A TEM instrument (Hitachi, Tokyo, Japan) was then applied to evaluate the samples.

### In vitro release study of antagomir-182-5p from CS-NPs formulation

CS-NPs-antagomir-182-5p were suspended within 1 ml of Tris-EDTA buffer (TE buffer) in RNase free Eppendorf tubes an incubated at 37 °C with shaking at 60 rpm as previous description [37]. At appointed time intervals, the supernatant was harvested for evaluation and renewed with new buffer. Quant-iT RiboGreen RNA Assay Kit was employed to determine the quantity of released antagomir-182-5p.

### Gel retardation assay

As for antagomir-182-5p integrity, CS-NPs were mixed with loading buffer and loaded on a 2% agarose gel for electrophoresis. CS-NPs and antagomir-182-5p were pretreated with or without RNases and then mixed with loading buffer and loaded on a 2% agarose gel for electrophoresis. The Bio-rad gel documentation system was used to visualize antagomir-182-5p integrity.

### In vitro uptake of CS-NPs

The FITC-labeled antagomir-182-5p were purchased from General BioL (Chuzhou, China) and encapsulated into CS-NPs as above mentioned. U251-MG cells were planted at a density of 5,000 cells/well on 96-well plates within culture medium, followed by overnight incubation at 37 °C. Cells were grown to 75% confluence, cells were subjected to treatment with CS-NPs(antagomir-182-5p)-FITC and antagomir-182-5p-FITC with the help of transfection reagent (Invitrogen). Twenty-four h later, U251-MG cells were observed under a fluorescent microscope (Olympus, Tokyo, Japan). For miR-182-5p and CDKN2C level determination, U251-MG cells were transfected with CS-NPs(antagomir-182-5p), negative control miRNA-loaded CS-NPs(CS-NPs (antagomir-NC)) or same amount of antagomir-182-5p for 48 h and then collected for qRT-PCR or westernblot analysis.

### Statistical analysis

GraphPad Prism (La Jolla, USA) was applied to analyze the findings of at least three different experiments, and the results were presented in form of mean ± standard deviation (SD). To determine statistical significance, a one-way analysis of variance (ANOVA) and Tukey’s multiple comparison test, or Student’s t-test were carried out to process data. Correlation analysis was performed according to Pearson’s correlation of all data generated in the present study. The significance level was set at *P* < 0.05.

## Results

### miR-182-5p is up-regulated in *IDH1* -mutant glioma samples

**Fig. 1:**
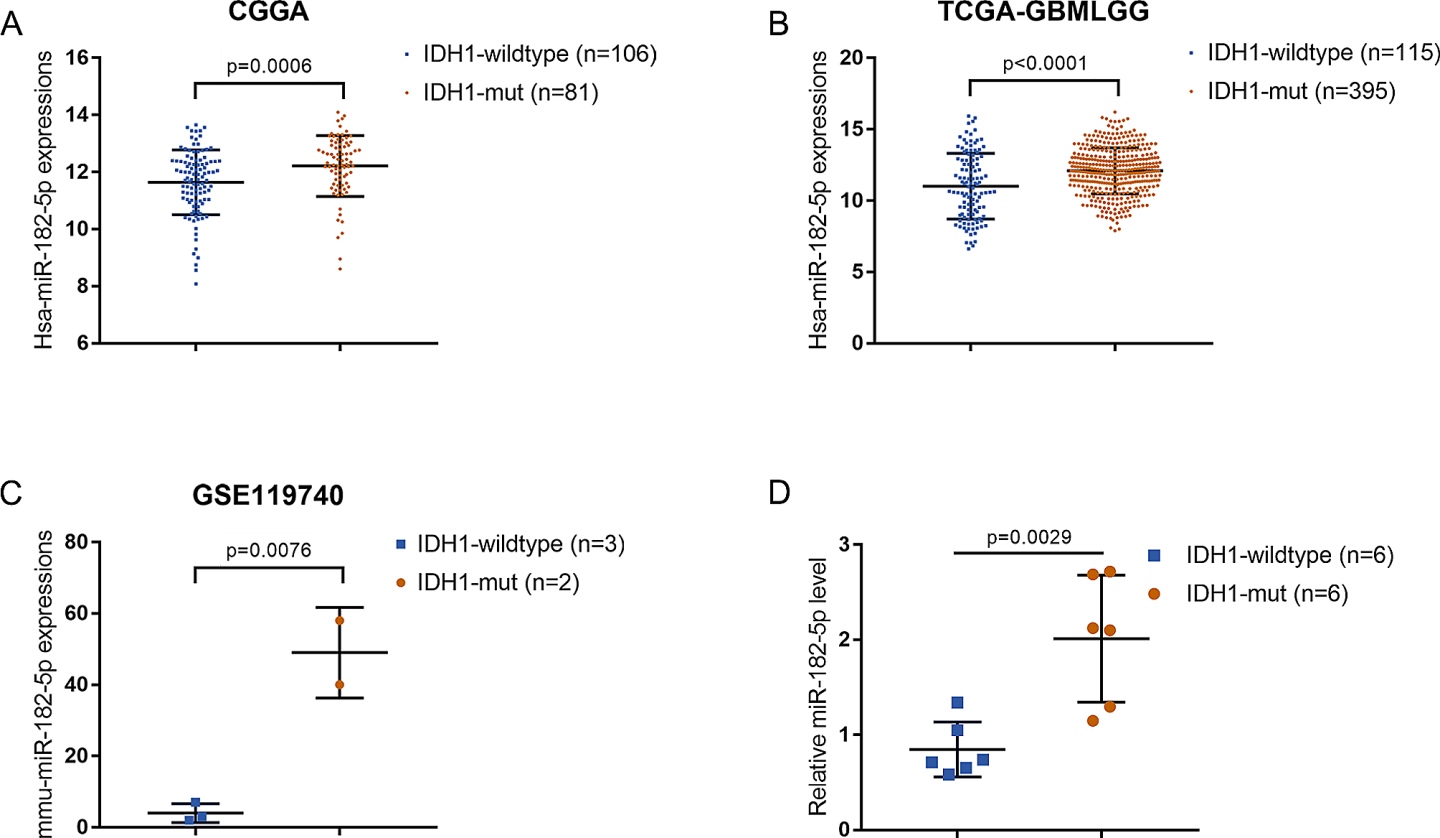
miR-182-5p is up-regulated in *IDH1* -mutant glioma samples. The expression levels of miR-182-5p in *IDH1*-mutant and *IDH1*-wildtype glioma samples according to CGGA database (**A**); TCGA-GBMLGG (**B**); GSE119740 (**C**); qRT-PCR analysis on collected clinical samples (**D**)

To identify differentially miRNAs that might be involved in *IDH1*-mutant gliomas, three online datasets, CGGA, TCGA-GBMLGG, and GSE119740 were analyzed. The miRNA microarray for 198 samples from CGGA (CGGA microRNA_array_198_gene), covering 829 miRNA expression information, was used. Among them, 187 cases were with *IDH1* mutation information, including 81 cases of *IDH1*-mutant and 106 cases of *IDH1*-wildtype, and 11 cases without *IDH1* mutation information were excluded. After processing by R language limma package, 150 up-regulated and 153 down-regulated miRNAs in *IDH1*-mutant samples were obtained (logFC > 0.4 or <-0.4, adjusted *P*. value < 0.05; Fig. S1A). TCGA-GBMLGG dataset included miRNA expression information in 115 *IDH1*-wildtype and 397 *IDH1*-mutant glioma samples. After data processing, 20 up-regulated and 288 down-regulated miRNAs in *IDH1*-mutant samples were obtained (logFC > 0.4 or <-0.4, adjusted *P*. value < 0.05; Fig. S1B). GSE119740 contained miRNA that were changed in glioma initiating cells after *IDH1* mutation. Data processing yielded 14 down-regulated and 19 up-regulated miRNAs in *IDH1*-mutant samples (logFC > 0.4 or <-0.4, adjusted *P*. value; Fig. S1C). Then, by cross-comparing the miRNAs that were significantly differentially up-regulated in IDH1-mutant samples in the three databases (CGGA, TCGA-GBMLGG and GSE119740), miR-182-5p was obtained (Fig. S1D). Moreover, the specific effect of miR-182-5p upon *IDH1*-mutant gliomas remains unclear. Therefore, miR-182-5p was selected for follow-up research focus. According to CGGA (Fig. 1A), TCGA-GBMLGG (Fig. 1B), and GSE119740 (Fig. 1C), miR-182-5p expression was significantly upregulated within *IDH1*-mutant glioma cells. According to qRT-PCR analysis, a significant upregulation has been found in miR-182-5p expression within collected *IDH1*-mutant glioma samples (Fig. 1D). In summary, miR-182-5p might contribute to *IDH1*-mutant glioma development.

### Mir-182-5p mediates R-2HG effects on glioma cell malignant behaviors

**Fig. 2:**
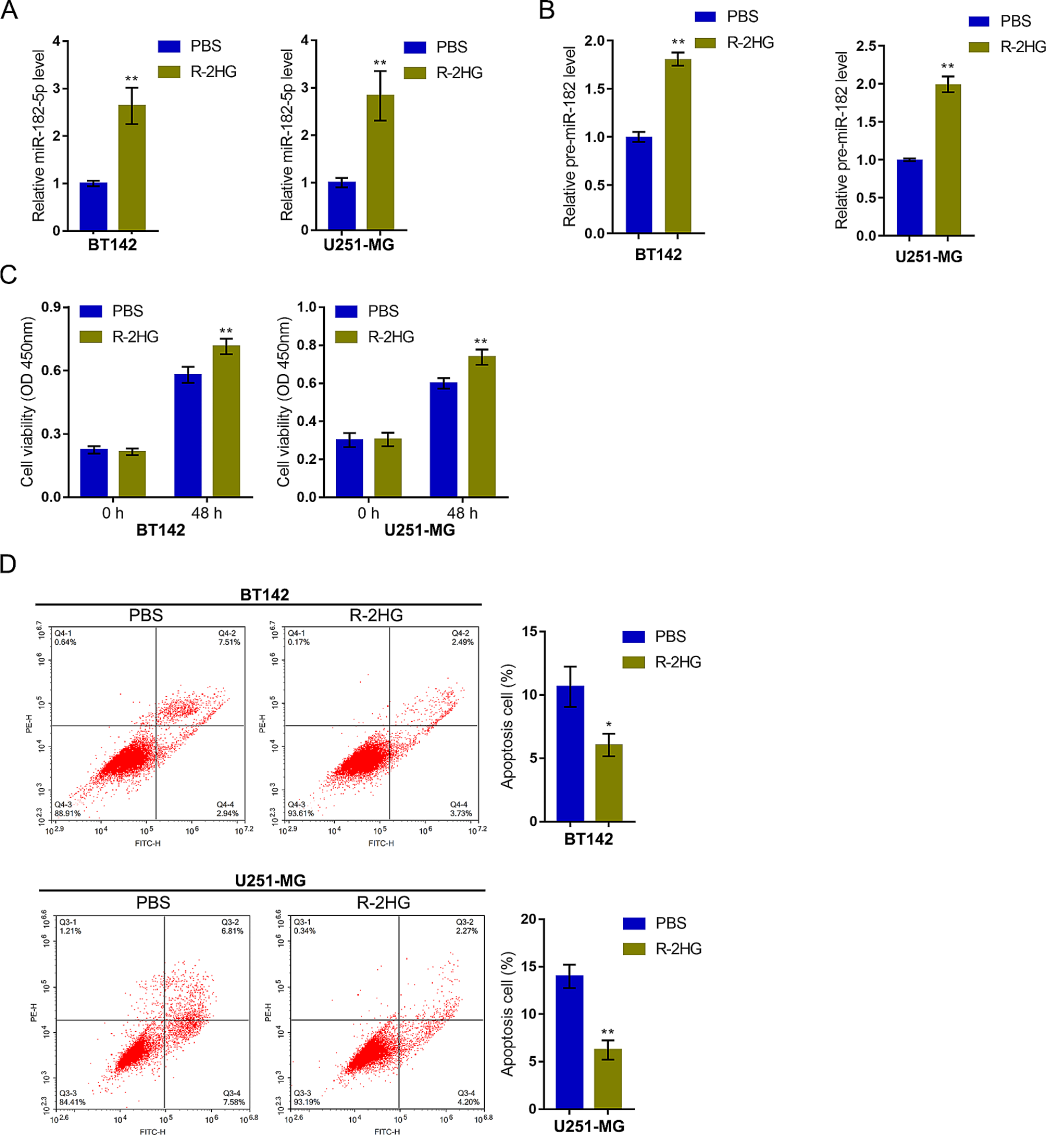
R-2HG up-regulates miR-182-5p expression and facilitates glioma cell malignant phenotypes. Glioma cell lines BT142 and U251-MG were treated with R-2HG and examined for mature miR-182-5p (**A**) and pre-miR-182-5p (**B**) expression using qRT-PCR; cell viability using a CCK-8 kit (**C**); cell apoptosis using Flow cytometry (**D**). *p < 0.05, ** *p* < 0.01 compared to PBS group

As aforementioned, mutations in *IDH1* produced elevated levels of R-2HG, which was found to be an oncometabolite [15, 18, 35]. Therefore, the effects of R-2HG upon miR-182-5p expression and *IDH1* mutant glioma cell phenotype were investigated using glioma cell lines BT142 and U251-MG. Under R-2HG treatment, the expression level of miR-182-5p showed to be markedly upregulated within both cell lines (Fig. 2A). Moreover, pre-miR-182-5p levels were also upregulated by R-2HG in glioma cells (Fig. 2B). Meanwhile, R-2HG treatment significantly promoted viability (Fig. 2C) and inhibited apoptosis of glioma cells (Fig. 2D).

**Fig. 3:**
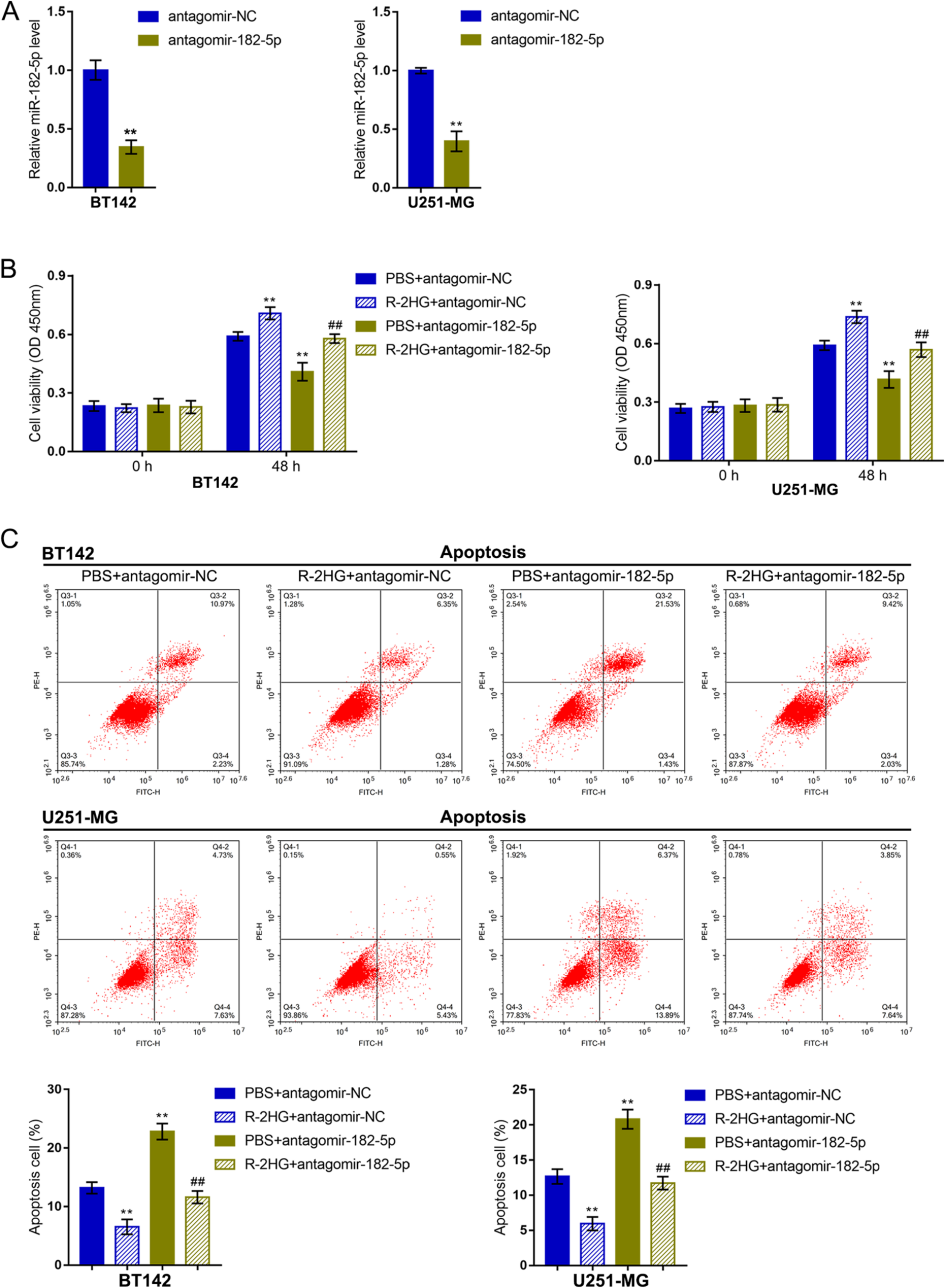
miR-182-5p mediates R-2HG effects on glioma cell malignant behaviors. (**A**) miR-182-5p inhibition was achieved in glioma cell lines BT142 and U251-MG by transducing antagomir-182-5p and confirmed using qRT-PCR. Then, BT142 and U251-MG were transduced with antagomir-182-5p, treated with R-2HG, and examined for cell viability using a CCK-8 kit (**B**); cell apoptosis using Flow cytometry (**C**). ** *p* < 0.01 compared with PBS + antagomir-NC; ## *p* < 0.01, compared with PBS + antagomir-182-5p

Secondly, the specific effects of miR-182-5p upon R-2HG oncogenic functions on glioma cells were investigated. miR-182-5p inhibition was achieved within glioma cell lines BT142 and U251-MG by transducing antagomir-182-5p and confirmed using qRT-PCR (Fig. 3A). Then, BT142 and U251-MG were transduced with antagomir-182-5p, stimulated with R-2HG, and determined for cell phenotypes. miR-182-5p inhibition significantly attenuated R-2HG-induced increase in viability (Fig. 3B) and inhibition upon cell apoptosis (Fig. 3C); in other words, miR-182-5p inhibition suppressed viability and enhanced apoptosis of glioma cells upon R-2HG treatment (Fig. 3B-C).

### In vivo functions of miR-182-5p and R-2HG in mice model

**Fig. 4:**
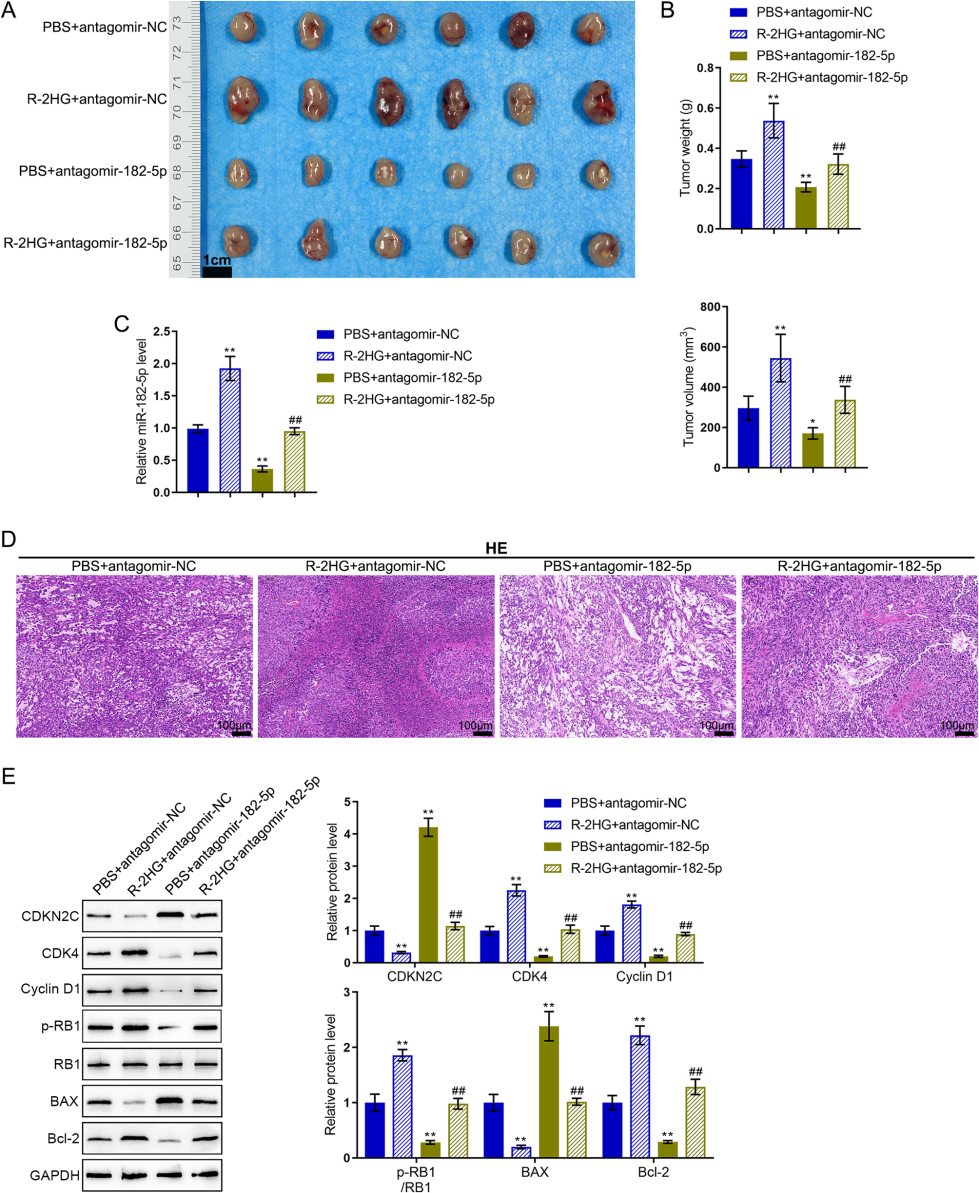
In vivo functions of miR-182-5p and R-2HG in mice model. (**A-B**) Xenograft tumor model was established in nude mice and R-2HG and/or antagomir-182-5p injection was administered as described. Tumor appearance, weight, and volume were examined. Scale bar = 1 cm. (**C**) The miR-182-5p levels in tumor tissues were examined using qRT-PCR. (**D**) Pathological alterations of tumor tissues were evaluated using H&E staining. Scale bar = 100 μm. (**E**) The protein levels of CDKN2C, CDK4, Cyclin D1, p-RB1, RB1, Bax, and Bcl-2 in tumor tissues were examined using Immunoblotting. * *p* < 0.05, ** *p* < 0.01 compared with PBS + antagomir-NC; ## *p* < 0.01, compared with PBS + antagomir-182-5p

As for the in vivo effects of miR-182-5p, xenograft tumor model was established in nude mice and R-2HG and/or antagomir-182-5p injection was administered as described. Figure 4A-B showed that R-2HG dramatically enlarged tumors, increased tumor weight, and increased tumor volume, while miR-182-5p knockdown remarkably reduced tumors, elevated tumor weight, and boosted tumor volume; when co-transduced, miR-182-5p knockdown partially relieved the tumor-promoting effects of R-2HG. In tumor tissues, the miR-182-5p levels showed to be considerably increased by R-2HG, downregulated by antagomir-182-5p; when co-transduced, the promotive effects of R-2HG on miR-182-5p levels were partially abolished via antagomir-182-5p (Fig. 4C). H&E staining was performed to evaluate the pathological alterations of tumor tissue samples (Fig. 4D). Compared to the model control group, R-2HG elevated mitotic activity and vascular proliferation and promoted cell density in tumor tissues. While there was tumor necrosis and a decrease in cell density and changes in morphology, with irregular cell shapes and dispersed, light purple and unevenly colored nuclei in antagomir-182-5p treatment mice tumor tissues (Fig. 4D). Immunoblotting was conducted to examine CDKN2C, CDK4, Cyclin-D1, p-RB1, RB1, Bax, and Bcl-2 protein contents within tumor tissue samples. Figure 4E shows that R-2 HG remarkably reduced CDKN2C and Bax protein contents, whereas increased Cyclin-D1, CDK4, RB1 phosphorylation and Bcl-2 proteins; miR-182-5p knockdown exerted opposite effects upon these proteins, and partially alleviated the effects of R-2HG.

### miR-182-5p directly binds to *CDKN2C* and inhibits its expression

**Fig. 5:**
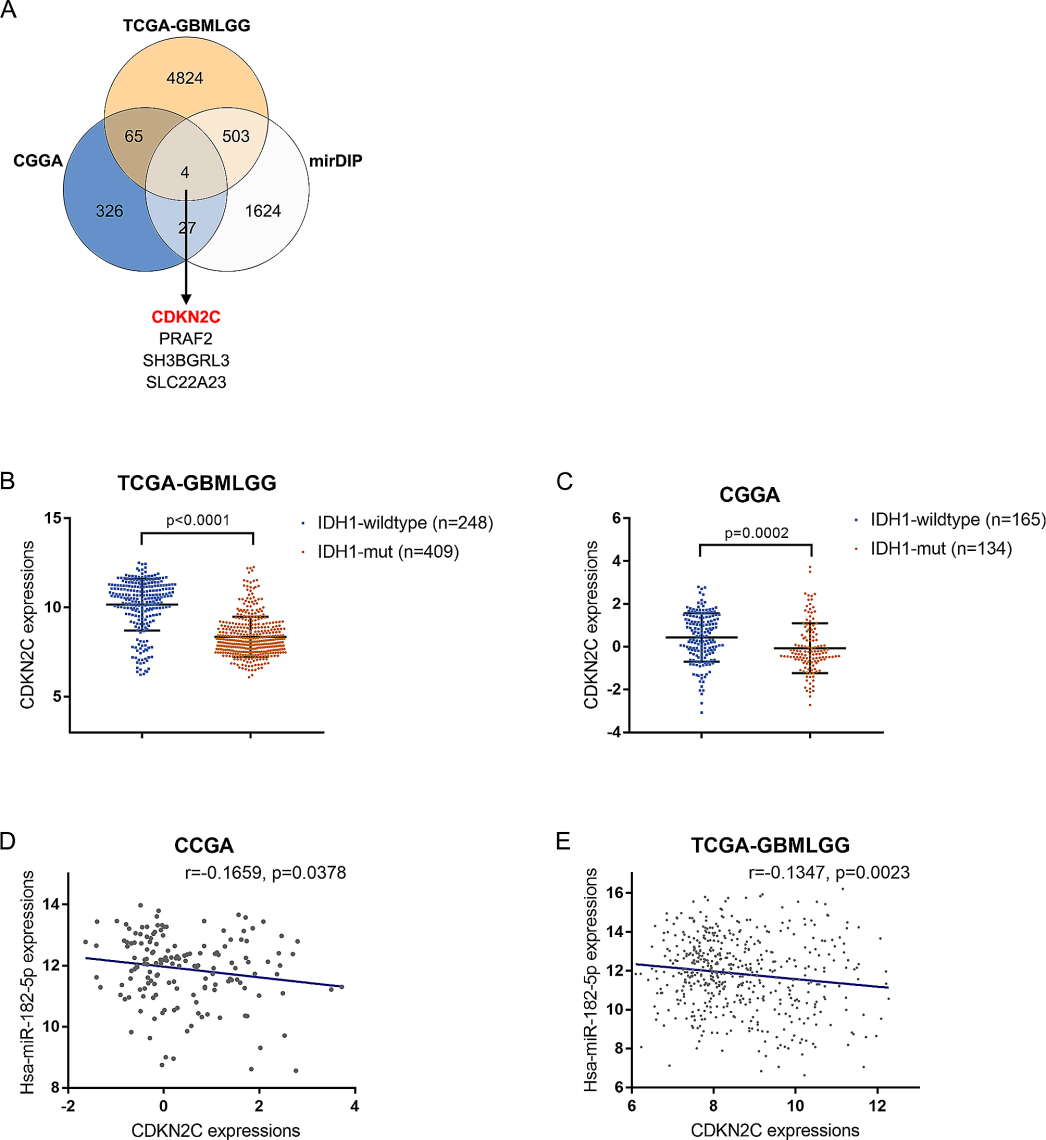
Analyzing downstream targets of miR-182-5p. (**A**) Genes negatively correlated with miR-182-5p were analyzed using CGGA and TCGA-GBMLGG data; mirDIP was used to analyze possible downstream targets of miR-182-5p. Obtained genes were compared and four candidates were overlapped: *CDKN2C*, *PRAF2*, *SH3BGRL3*, and *SLC22A23*. (**B-C**) The expression levels of *CDKN2C* in *IDH1*-mutant and *IDH1*-wildtype glioma samples according to TCGA-GBMLGG and CGGA. (**D-E**) The correlation between miR-182-5p and *CDKN2C* analyzed using Pearson’s correlation analysis according to CGGA and TCGA-GBMLGG

Considering that miRNAs exert their physiological functions through binding and regulating downstream targets, miR-182-5p downstream targets were analyzed. Genes negatively correlated with miR-182-5p were analyzed using CGGA and TCGA-GBMLGG data; mirDIP, an online tool that help find genes targeted by a microRNA in Homo sapiens (https://ophid.utoronto.ca/mirDIP/) [36, 37] was utilized to analyze putative downstream targets of miR-182-5p. Obtained genes were compared and four candidates were overlapped: *CDKN2C*, *PRAF2*, *SH3BGRL3*, and *SLC22A23* (Fig. 5A). CDKN2C (cell cycle protein-dependent kinase inhibitor 2 C, also known as p18) inhibits CDK4 and CDK6 activation and negatively regulates cell cycle, acting as a tumor suppressor. Missense mutations, nonsense mutations, silent mutations, and shift deletions and insertions in *CDKN2C* have been found in cancer patients, including gliomas [38–40]. Therefore, *CDKN2C* was selected for further investigations. According to TCGA-GBMLGG and CGGA, *CDKN2C* expression was remarkably decreased in *IDH1*-mutant glioma samples than those in *IDH1*-wildtype glioma samples (Fig. 5B-C). Within tissues, miR-182-5p exhibited a negative correlation with *CDKN2C*, according to TCGA-GBMLGG and CGGA (Fig. 5D-E).

**Fig. 6:**
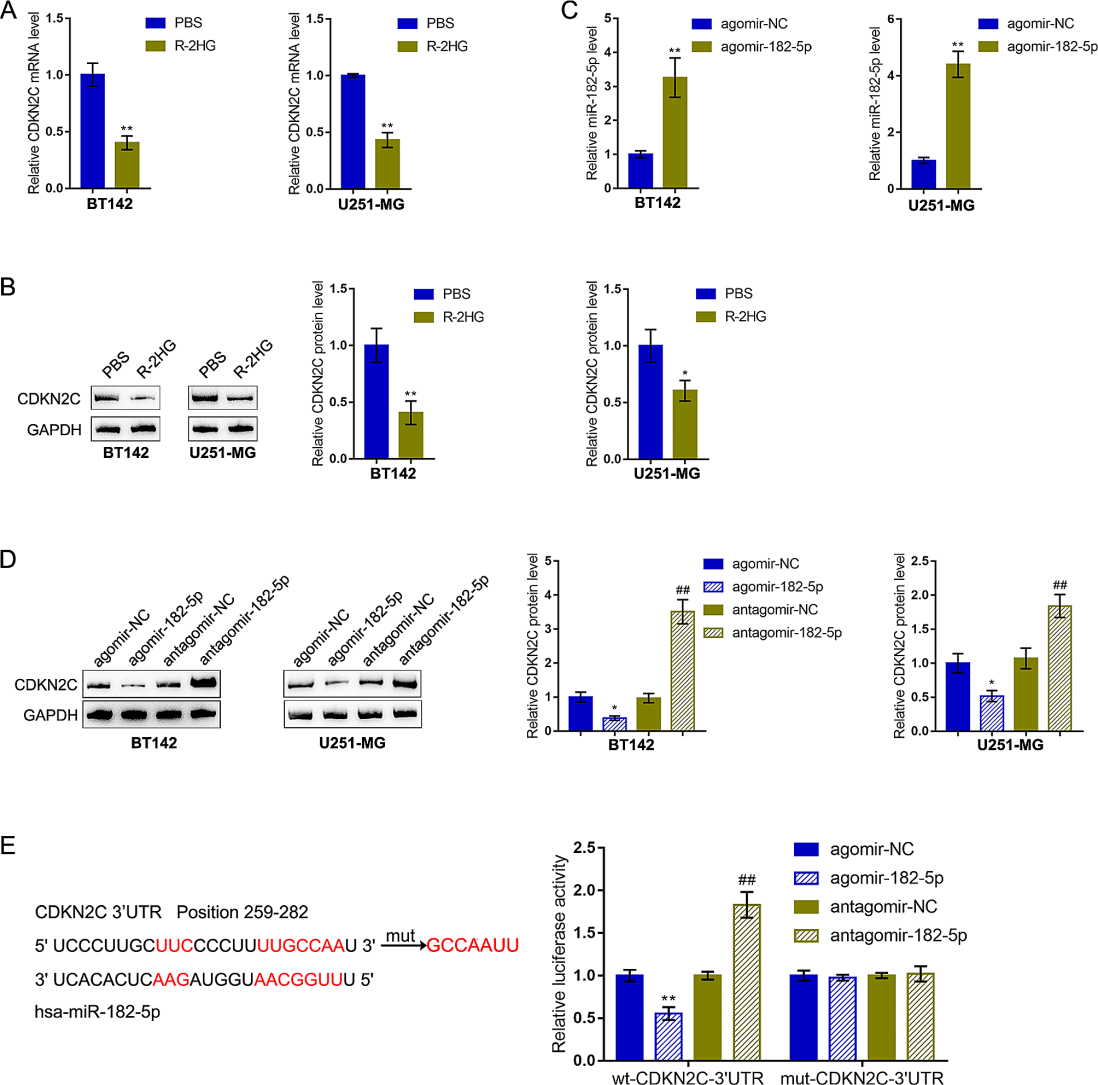
miR-182-5p directly targets *CDKN2C* and inhibits its expression. (**A-B**) Glioma cell lines BT142 and U251-MG were treated with R-2HG and examined for *CDKN2C* mRNA expression and protein levels using qRT-PCR and Immunoblotting, respectively. (**C**) miR-182-5p overexpression was achieved in BT142 and U251-MG cells by transducing agomir-182-5p and confirmed using qRT-PCR. (**D**) BT142 and U251-MG cells were transduced with antagomir-182-5p or agomir-182-5p and examined for the protein levels of CDKN2C using Immunoblotting. (**E**) *CDKN2C* 3’UTR-wt and *CDKN2C* 3’UTR-mut reporters were constructed based on psiCheck-2 plasmid and co-transduced into tool cells with antagomir-182-5p or agomir-182-5p; the luciferase activity was determined. * *p* < 0.05, ** *p* < 0.01 compared with PBS or agomir-NC; ## *p* < 0.01, compared with antagomir-NC

Furthermore, in BT142 and U251-MG glioma cell lines treated with R-2HG, *CDKN2C* mRNA expression and protein levels were significantly decreased (Fig. 6A-B). Fore investigating miR-182-5p regulation of *CDKN2C*, agomir-182-5p was transduced by achieving miR-182-5p overexpression within BT142 and U251-MG cells, as verified by qRT-PCR (Fig. 6C). Within BT142 and U251-MG cell lines, miR-182-5p overexpression reduced, while miR-182-5p knockdown increased CDKN2C protein contents (Fig. 6D). For validating the direct binding of miR-182-5p to *CDKN2C*, *CDKN2C* 3’UTR-wt and *CDKN2C* 3’UTR-mut reporters were constructed based on psiCheck-2 plasmid and co-transduced into tool cells with antagomir-182-5p or agomir-182-5p; the luciferase activity was examined. While co-transducing with *CDKN2C* 3’UTR-wt, miR-182-5p overexpression significantly inhibited, whereas miR-182-5p knockdown promoted *CDKN2C* 3’UTR-wt luciferase activity; while co-transducing with *CDKN2C* 3’UTR-mut, miR-182-5p overexpression/inhibition failed to alter *CDKN2C* 3’UTR-mut luciferase activity (Fig. 6E). In summary, miR-182-5p directly binds *CDKN2C* and represses *CDKN2C* expression.

### Dynamic effects of the miR-182-5p/ *CDKN2C* axis on R-2HG-induced malignant cell phenotypes

**Fig. 7:**
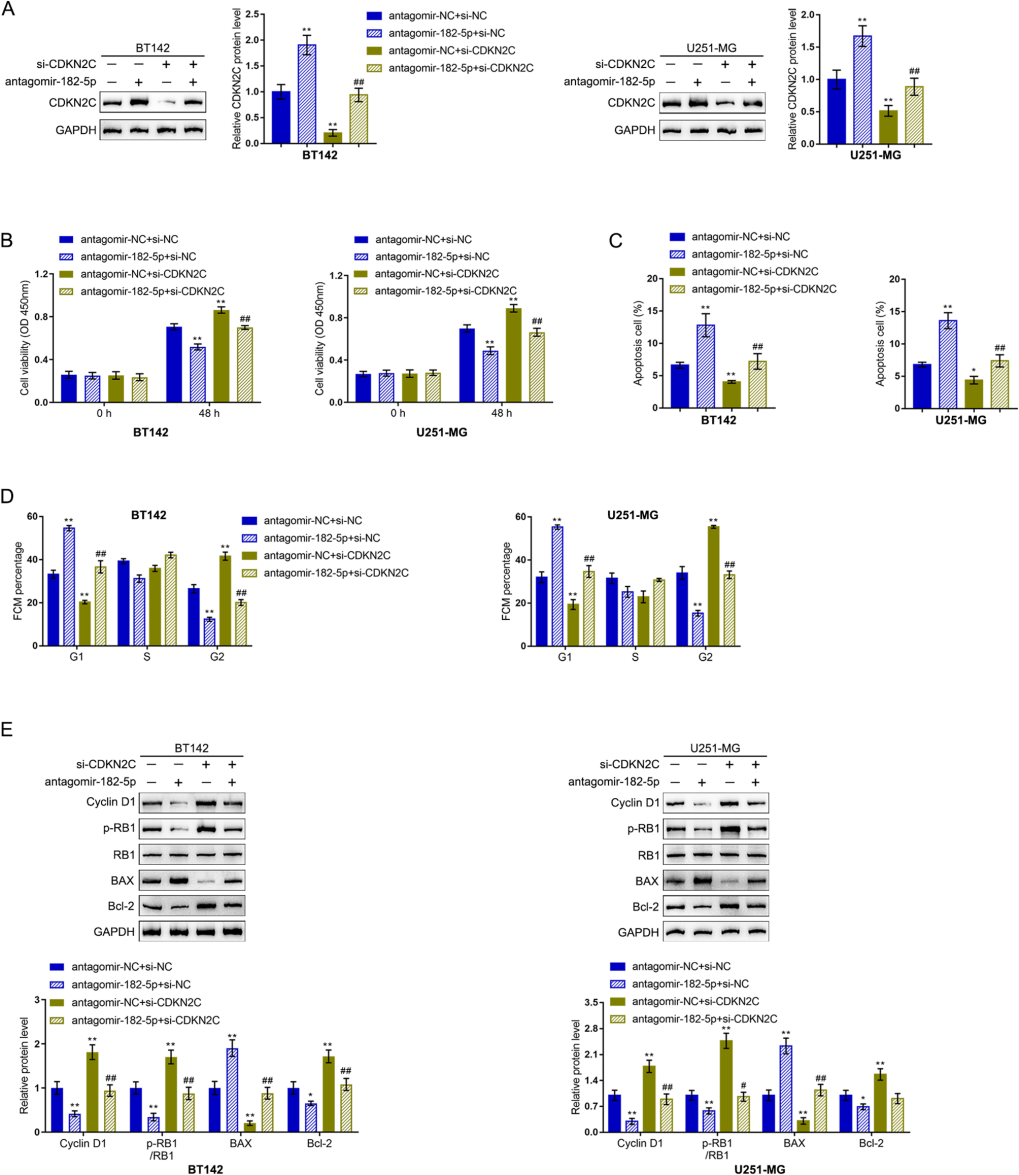
Dynamic effects of the miR-182-5p/CDKN2C axis on R-2HG-induced malignant cell phenotypes. BT142 and U251-MG cells were co-transduced with antagomir-182-5p and small interfering RNA targeting CDKN2C (si-CDKN2C), treated with R-2HG, and examined for the protein levels of CDKN2C using Immunoblotting (**A**); cell viability using CCK-8 assay (**B**); cell apoptosis and cell cycle distribution using Flow cytometry (**C-D**); the protein levels of cyclin D1, p-RB1, RB1, Bax, and Bcl-2 using Immunoblotting (**E**). * *p* < 0.05, ** *p* < 0.01 compared with antagomir-NC + si-NC; ## *p* < 0.01, compared with antagomir-NC + si- CDKN2C

Last, the dynamic effects of the miR-182-5p/*CDKN2C* axis upon the phenotypes of R-2HG-triggered malignant cells were investigated. BT142 and U251-MG cells were co-transduced with antagomir-182-5p and si-*CDKN2C*, treated with R-2HG, and determined for CDKN2C protein contents. Under R-2HG treatment, miR-182-5p knockdown increased, whereas si-*CDKN2C* decreased CDKN2C protein levels; the effects of antagomir-182-5p upon CDKN2C protein levels were partially attenuated by si-*CDKN2C* (Fig. 7A). Regarding cellular phenotypes, under R-2HG treatment, miR-182-5p inhibition suppressed cell viability, enhanced cell apoptosis, and triggered G1-phase arrest of cell cycle; in contrast, *CDKN2C* knockdown exerted the opposite effects (Fig. 7B-D and Fig. S2). More importantly, *CDKN2C* knockdown partially attenuated the effects of miR-182-5p inhibition on cell phenotypes (Fig. 7B-D). Regarding the cell cycle check point and apoptosis markers, cyclin D1, and Bcl-2 protein contents and RB1 phosphorylation were decreased and Bax was increased by miR-182-5p inhibition; in contrast, *CDKN2C* knockdown increased cyclin D1, and Bcl-2 protein contents and promoted RB1 phosphorylation, whereas decreased Bax protein levels. Moreover, *CDKN2C* knockdown also significantly relieved the effects of miR-182-5p knockdown upon these markers (Fig. 7E). These findings indicate that R-2HG exerts its oncogenic effects on glioma cells via the miR-182-5p/*CDKN2C* axis.

### In vivo antitumor effects of CS-NPs (antagomir-182-5p) on xenograft mouse models

**Fig. 8:**
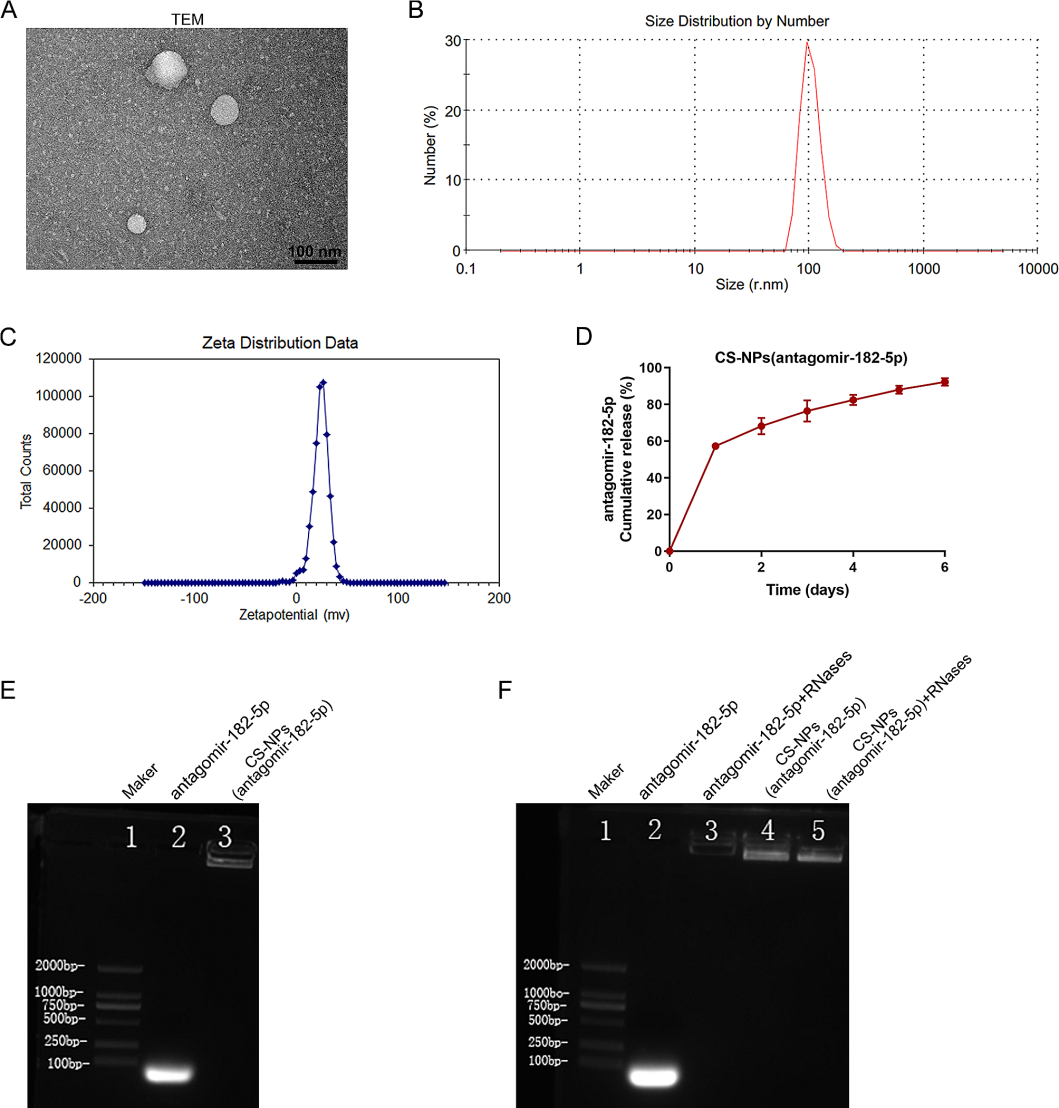
Character of CS-NPs(antagomir-182-5p). (**A**) Electron microscope image of CS-NPs(antagomir-182-5p). Scale bar = 100 nm. (**B-C**) The size range and zeta potential of CS-NPs(antagomir-182-5p). (**D**) In vitro release profile of antagomir-182-5p from CS-NPs(antagomir-182-5p). (**E-F**) Confirmation of antagomir-182-5p encapsulation into CS-NPs by gel retardation assay

CS-NPs seem a very promising strategy that has been utilized for gene delivery including miRNAs in GBM [41, 42]. Therefore, we further investigated the antitumor effects of CS-NPs (antagomir-182-5p) on xenograft mouse models. Firstly, CS-NPs (antagomir-182-5p) were spontaneously formed, and then separated by centrifugation. TEM (Fig. 8A) was employed to examine the morphological changes of CS-NPs (antagomir-182-5p), and solid, consistent, and compact structures with spherical shapes were revealed. Zetasizer, Nano ZS (Fig. 8B-C) was utilized to perform the size and zeta potential analysis. CS-NPs(antagomir-182-5p) were synthesized with the size of 100 nm, polydispersity index (PDI) of 0.25 and zeta potential of + 24 mV. In vitro release profile of antagomir-182-5p from CS-NPs (antagomir-182-5p) shows a stable controlled-release of antagomir-182-5p (Fig. 8D). Gel retardation assay results showed that the band for antagomir-182-5p under agarose nucleic acid electrophoresis is normally below 50 bp. The lane for CS-NPs (antagomir-182-5p) remained stationary, with the sample stagnating at the loading well (Fig. 8E). The ribonuclease protection assay demonstrated that after treatment with RNases, both antagomir-182-5p and CS-NPs (antagomir-182-5p) underwent agarose nucleic acid electrophoresis. The results indicated that the nanomaterial CS-NPs (antagomir-182-5p) can protect antagomir-182-5p from degradation by RNA nucleases (Fig. 8F).

**Fig. 9:**
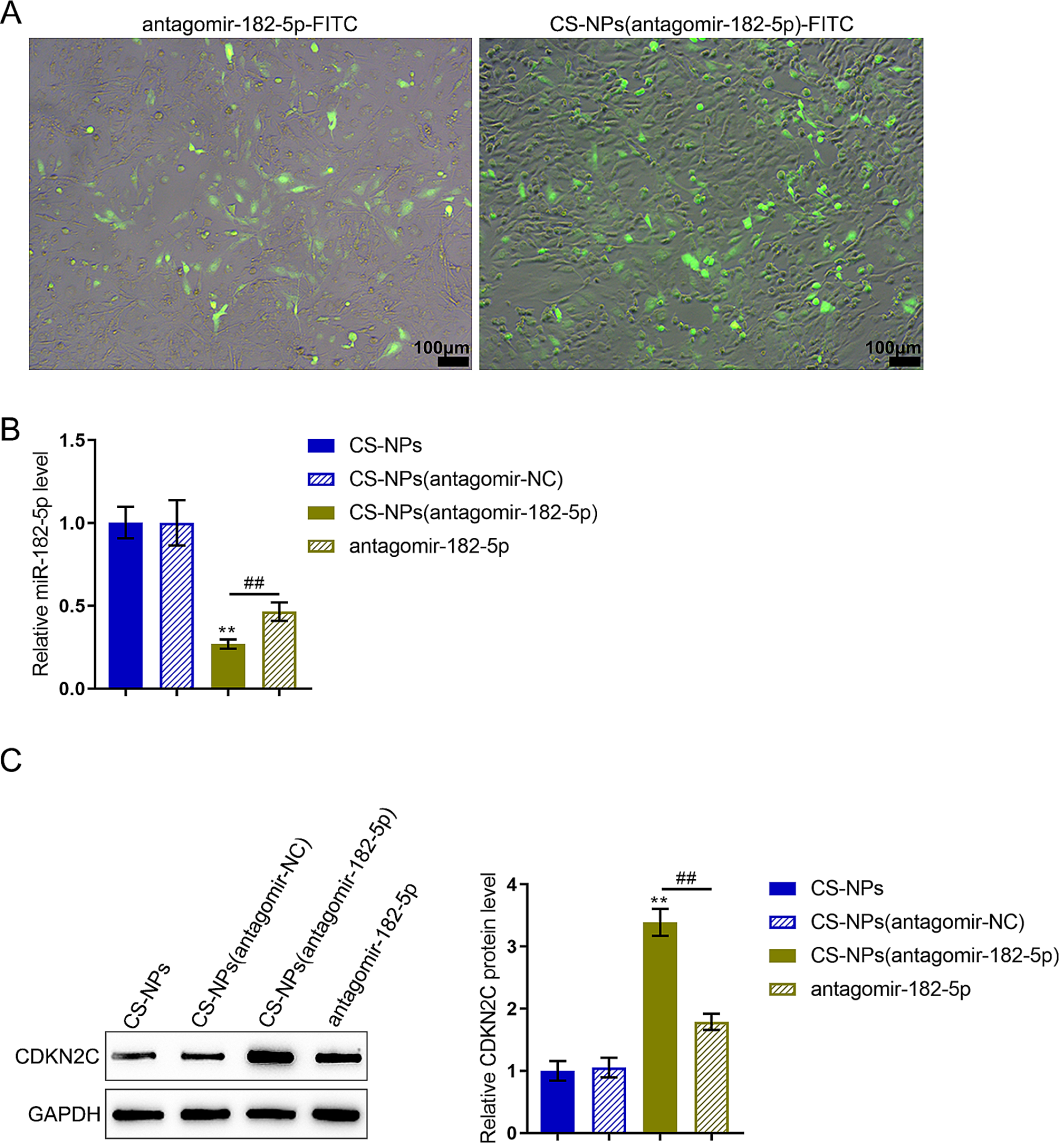
Transfection efficiency of CS-NPs(antagomir-182-5p). (**A**) Transfection efficiency of FITC-expressing antagomir-182-5p and CS-NPs(antagomir-182-5p) in U251-MG cells after 48 h. Scale bar = 100 μm. (**B**) The expression levels of miR-182-5p in U251-MG cells transfected with empty CS-NPs, CS-NPs(antagomir-NC), CS-NPs(antagomir-182-5p), or antagomir-182-5p were examined using qRT-PCR. (**C**) The protein levels of CDKN2C levels in U251-MG cells transfected with empty CS-NPs, CS-NPs(antagomir-NC), CS-NPs(antagomir-182-5p), or antagomir-182-5p were examined using Immunoblotting. ** *p* < 0.01 compared with CS-NPs(antagomir-NC); ## *p* < 0.01, compared with antagomir-182-5p

Next, the effects of CS-NPs upon the expression of miR-182-5p and it’s target *CDKN2C* were examined. As shown by fluorescence, CS-NPs (antagomir-182-5p)-FITC was successfully transfected in U251-MG cells after 24 h (Fig. 9A). miR-182-5p expression levels were downregulated within U251-MG cells transfected with CS-NPs (antagomir-182-5p) and antagomir-182-5p compared with the empty CS-NPs and CS-NPs(antagomir-NC) groups; CS-NPs(antagomir-182-5p) downregulated miR-182-5p more (Fig. 9B). The protein contents of CDKN2C, the target gene of miR-182-5p, were elevated in U251-MG cells transfected with CS-NPs(antagomir-182-5p) and antagomir-182-5p compared with the empty CS-NPs and CS-NPs(antagomir-NC) groups; CS-NPs(antagomir-182-5p) increased CDKN2C protein levels more (Fig. 9C).

**Fig. 10:**
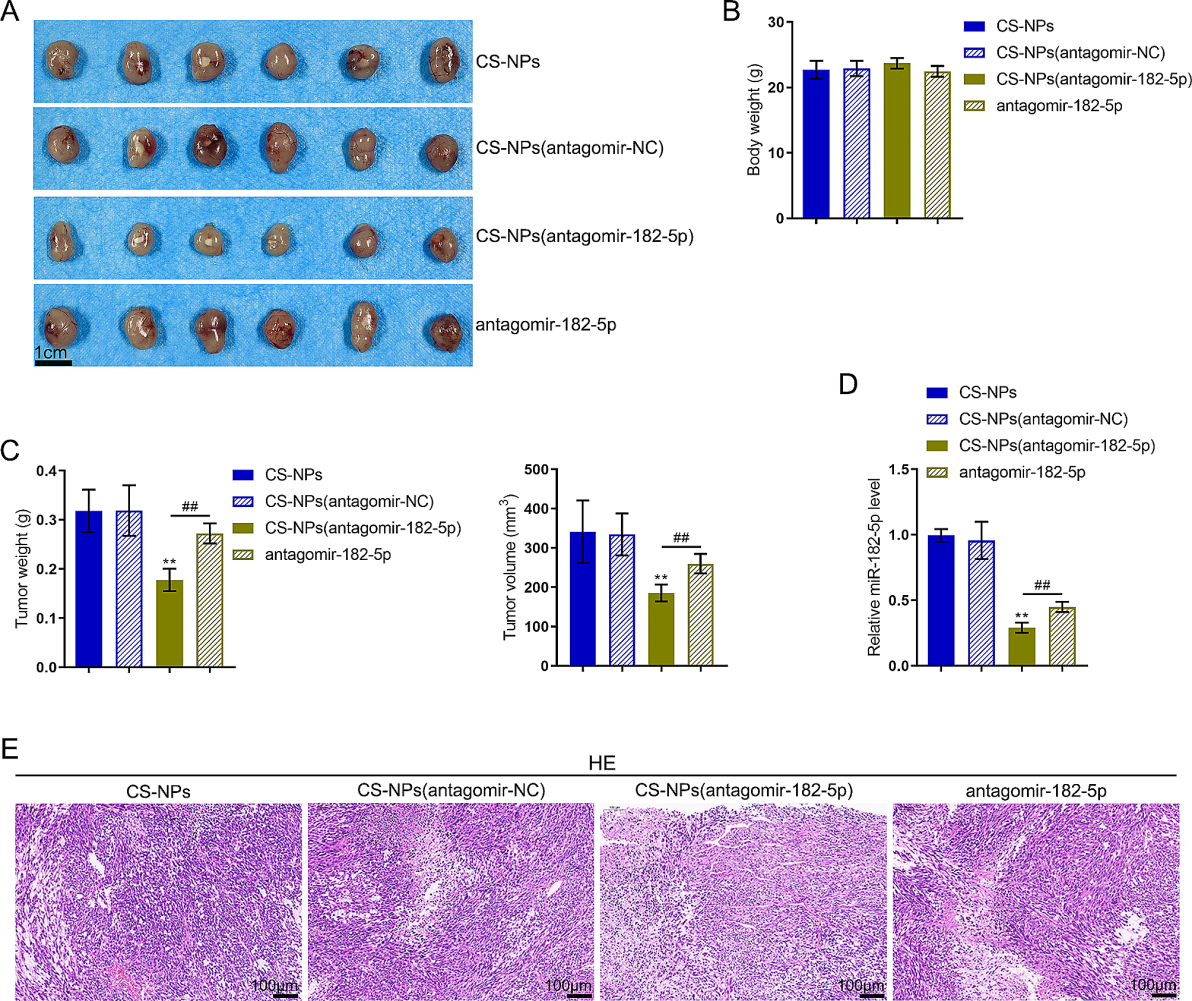
In vivo antitumor effects of CS-NPs(antagomir-182-5p) on xenograft mouse models. (**A-C**) Xenograft tumor model was established in nude mice and antagomir-182-5p or CS-NPs(antagomir-182-5p) injection was administered as described. Scale bar = 1 cm. Tumor appearance, body weight, tumor weight, and tumor volume were examined. (**D**) The miR-182-5p levels in tumor tissues were examined using qRT-PCR. (**E**) Pathological alterations of tumor tissues were evaluated using H&E staining. Scale bar = 100 μm. ** *p* < 0.01 compared with CS-NPs(antagomir-NC); ## *p* < 0.01, compared with antagomir-182-5p

As for the in vivo effects of CS-NPs (antagomir-182-5p), xenograft tumor model was constructed in nude mice and antagomir-182-5p or CS-NPs (antagomir-182-5p) intratumor injection was administered as described. CS-NPs biosafety was evaluated using H&E staining for pathological alterations. Fig.S3 shows that no obvious lesions on the liver or heart were observed. As shown in Fig. 10A-C, CS-NPs(antagomir-182-5p) and antagomir-182-5p both significantly decreased tumor weight and tumor volume without affecting mice body weight, whereas CS-NPs(antagomir-182-5p) exerted better effects decreasing tumor weight and tumor volume. Within tumor tissue samples, the miR-182-5p levels were significantly downregulated by CS-NPs(antagomir-182-5p) and antagomir-182-5p, whereas CS-NPs(antagomir-182-5p) downregulated miR-182-5p more (Fig. 10D). Pathological alterations of tumor tissues were evaluated using H&E staining (Fig. 10E). The number of tumor cells were significantly reduced, the tumor cell cytoplasm was ruptured, the nucleus was lysed, and the tumor cell necrosis area was larger in the tumor tissue of CS-NPs(antagomir-182-5p) and antagomir-182-5p treatment groups (Fig. 10E).

## Discussion

Herein, miR-182-5p expression showed to be increased within *IDH1*-mutant glioma tissue samples according to TCGA, CGGA, and online dataset GSE119740, as well as collected clinical samples. R-2HG treatment up-regulated the expression of miR-182-5p, enhanced glioma cell proliferation, and suppressed apoptosis; miR-182-5p knockdown partially eliminated R-2HG’s oncogenic effects upon glioma cells. By direct binding to *CDKN2C* 3’UTR, miR-182-5p inhibited *CDKN2* expression. Regarding cellular functions, *CDKN2C* knockdown promoted viability, suppressed apoptosis, and relieved cell cycle arrest of R-2HG-treated glioma cells. Furthermore, *CDKN2C* knockdown partially attenuated the functions of miR-182-5p knockdown upon cell phenotypes. Moreover, *CDKN2C* knockdown exerted opposite effects on cell cycle check point and apoptosis markers to those of miR-182-5p inhibition; also, *CDKN2C* knockdown partially attenuated the functions of miR-182-5p inhibition upon cell cycle check point and apoptosis markers. Herein, CS-NPs encapsulated antagomir-182-5p were synthesized and their underlying function as an anti-tumor agent against xenografted tumor in nude mice was explored. We showed that CS-NPs (antagomir-182-5p) had minimal toxicity in mice and exerted a favorable anti-tumor effect on xenograft tumor model in nude mice.

Both enantiomers of 2HG, R-2HG and S-2HG, have been found to be linked to tumor growth through their suppressive roles in αKG (α-ketoglutarate)-dependent dioxygenases. *IDH* mutations lead to a neomorphic enzymatic activity of the mutated IDH enzymes (mitochondrial IDH2 and cytosolic IDH1), from which the R-2HG is mainly derived, while S-2HG is generated as a result of pathological processes including hypoxia [43]. Although the role of R-2HG is complex and seemingly paradoxical, it has commonly been reported as an oncometabolite in gliomas. As aforementioned, AGI-5198 dose-dependently suppressed R-2HG production in mutant enzyme mIDH1. Under near-complete R-2HG inhibition conditions, inhibition of mIDH1 attenuated the proliferation of *IDH1*-mutated glioma cells without largely affecting genome-wide DNA methylation levels [18]. Herein, R-2HG treatment significantly induced malignant behaviors of glioma cells, as manifested as promoted cell viability and inhibited cell apoptosis. Notably, R-2HG significantly up-regulated miR-182-5p expression which was increased within *IDH1*-mutant glioma cells, suggesting the potential oncogenic effect of miR-182-5p on glioma.

miR-182-5p was considered to be an oncogenic miRNA within many malignancies, such as lung carcinoma [44], breast carcinoma [45], prostate carcinoma [46], liver carcinoma [47], colorectal carcinoma [48], and gliomas [49–52]. Reportedly, in glioma cells, miR-182-5p could affect the capacity of cancer cells to proliferate, invade and migrate, as well as the drug-sensitivity. Furthermore, miR-182-5p also enhances glioblastoma angiogenesis. Herein, after inhibiting miR-182-5p by antagomir-182-5p, R-2HG-induced glioma cell viability and R-2HG-suppressed cell apoptosis were partially reversed, confirming its oncogenic role in glioma. Mechanically, miRNAs play their roles via binding to downstream mRNAs [21]; herein, *CDKN2C* was considered to be a direct downstream target of miR-182-5p. Through direct binding to *CDKN2C* 3’UTR, miR-182-5p repressed the expression level of *CDKN2*. Conversely, *CDKN2C* expression was dramatically decreased in R-2HG-stimulated glioma cells, suggesting that *CDKN2C* might mediate the effects of miR-182-5p upon R-2HG-induced glioma cells.

Notably, *CDKN2C* belongs to the *INK4/CDKN2* family (*CDKN2A* [p15], *CDKN2B* [p16], *CDKN2C* [p18], and *CDKN2D* [p19]), which is one of the cyclin-dependent kinase inhibitors which suppress cell cycle development via the crosstalk with CDK4/6 to prevent cyclin D-CDK4/6 complex activation [53]. The abnormal CDKN loss resulted in uncontrolled RB phosphorylation and unregulated development via the S phase of the cell cycle, and was found to be associated with the progression of a variety of cancers [54–57]. Consistently, in this study, in addition to enhancing the cell viability and inhibiting cell apoptosis of R-2HG-treated glioma cells, *CDKN2C* knockdown also relived G1-phase arrest of cell cycle. As for cell cycle check point proteins and apoptotic signaling markers, *CDKN2C* knockdown increased CDK4, cyclin D1, and Bcl-2 protein contents and promoted RB1 phosphorylation, whereas decreased Bax protein levels. In contrast, miR-182-5p inhibition induced cell cycle arrest, as well as exerted opposite effects on cell cycle and apoptotic signaling markers. More importantly, *CDKN2C* knockdown partially abolished the tumor-suppressive roles of miR-182-5p inhibition, indicating that miR-182-5p exerts its functions upon R-2HG-treated glioma cells through *CDKN2C*. In vivo, miR-182-5p inhibition showed significant anti-tumor effects upon xenograft tumor model in nude mice, further confirming the anti-tumor effects of miR-182-5p inhibition.

Considering the excellent delivery properties of CS and NPs [58–60], we successfully synthesized CS-NPs(antagomir-182-5p) encapsulating antagomir-182-5p and explored their therapeutic potential against xenografted tumors in nude mice. Morphologically, these nanoparticles exhibited a consistent spherical shape, as observed under TEM, aligning with previous nanoparticle studies [61]. Their size, measured at 100 nm, and a polydispersity index (PDI) of 0.25, suggest a uniform distribution, which is crucial for consistent drug delivery [61]. The positive zeta potential of + 24 mV indicates stability in suspension, reducing the likelihood of aggregation [62]. Notably, the in vitro release profile demonstrated a controlled release of antagomir-182-5p, an essential feature for sustained therapeutic effects. Furthermore, the gel retardation assay and ribonuclease protection assay results collectively highlight the protective role of CS-NPs against RNA degradation, a challenge previously reported in naked RNA-based therapies [63]. The transfection efficiency results demonstrated the potential of CS-NPs(antagomir-182-5p) in downregulating miR-182-5p, resulting in an increase in its target protein, CDKN2C. This modulation is consistent with our in vitro results that miR-182-5p inhibition led to the increase in CDKN2C. In vivo, the CS-NPs (antagomir-182-5p) demonstrated safety, as evidenced by the absence of pathological alterations in vital organs and showcased superior anti-tumor effects compared to antagomir-182-5p alone. This potent anti-tumor activity, combined with the observed safety profile, positions CS-NPs (antagomir-182-5p) as a promising candidate for further therapeutic development against tumors. The enhanced anti-tumor effects of CS-NPs(antagomir-182-5p) might stem from the superior controlled-release properties of CS-NPs.

In conclusion, miR-182-5p is elevated while *CDKN2C* is diminished in *IDH1*-mutant gliomas and R-2HG-treated glioma cells. This miR-182-5p/*CDKN2C* dynamic drives the oncogenic effects of R-2HG, impacting cell viability, apoptosis, and the cell cycle. The crafted CS-NPs(antagomir-182-5p) adeptly encapsulate and deliver antagomir-182-5p, amplifying in vivo anti-tumor efficacy and in vivo safety in xenograft tumor model in mouse. Collectively, these insights highlight the potential of CS-NPs(antagomir-182-5p) to target the miR-182-5p/*CDKN2C* axis, offering a promising therapeutic avenue against R-2HG’s oncogenic influence in both cellular and mice models.

### Electronic supplementary material

Below is the link to the electronic supplementary material.

Supplementary Material 1

Supplementary Material 2

## Data Availability

Please contact the corresponding author for data requests.
