# Supplementary material for: IDH1 mutation produces R-2-hydroxyglutarate (R-2HG) and induces mir-182-5p expression to regulate cell cycle and tumor formation in glioma
Source: Biol Res. 2024 May 17;57:30. doi: 10.1186/s40659-024-00512-2 (PMC11100189; doi:10.1186/s40659-024-00512-2)
Supplement: Supplementary file 1 — Supplementary Material 1 [file 40659_2024_512_MOESM1_ESM.docx]

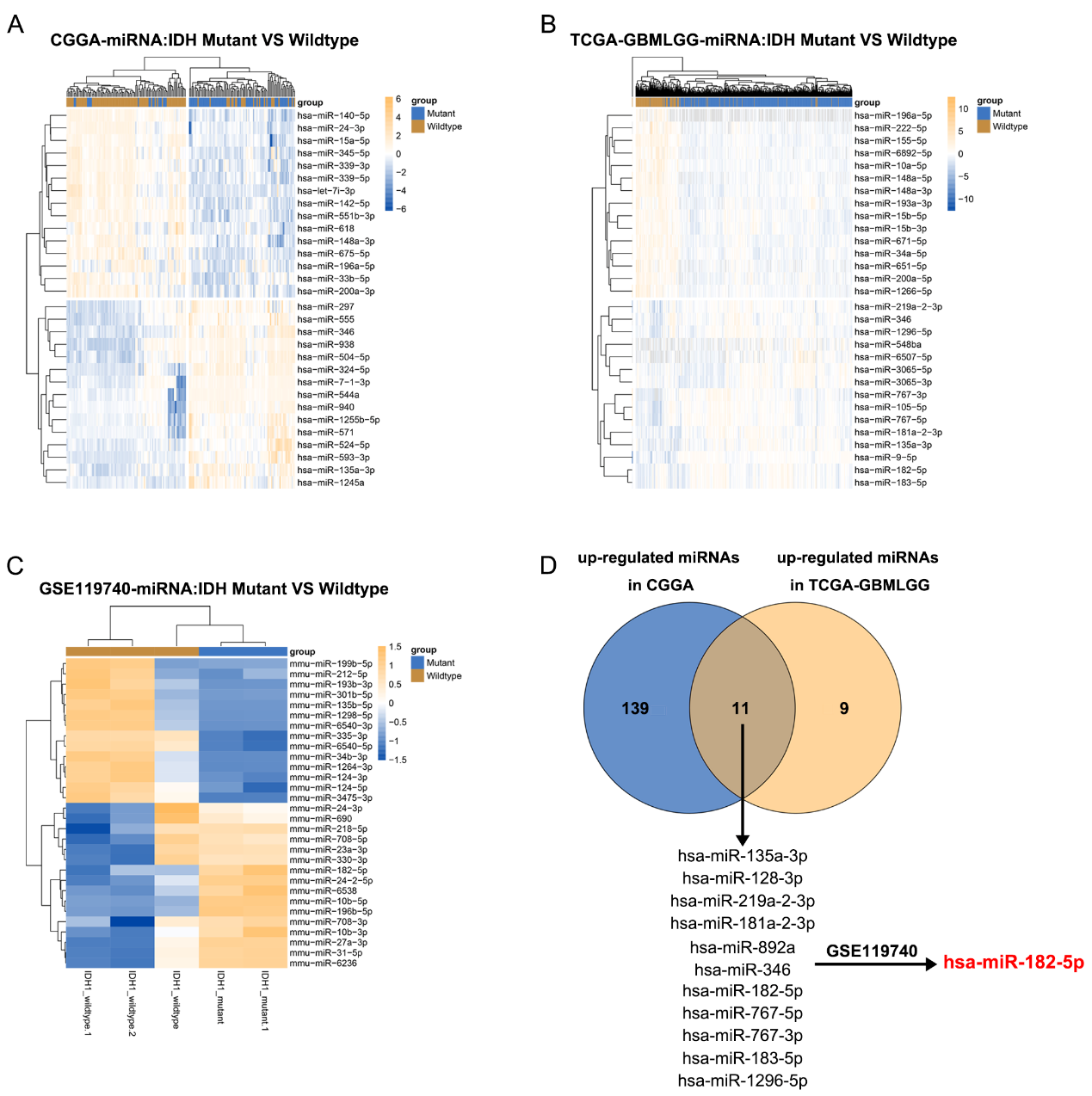


**Fig.S1 Differentially expressed miRNAs between IDH1-mutant and IDH1-wildtype glioma samples** (A-C) Hierarchical clustering showing differentially expressed miRNAs between IDH1-mutant and IDH1-wildtype glioma samples according to CGGA, TCGA-GBMLGG, and GSE119740. (D) Up-regulated miRNAs in IDH1-mutant glioma samples according to all the three datasets and miR-182-5p was obtained.


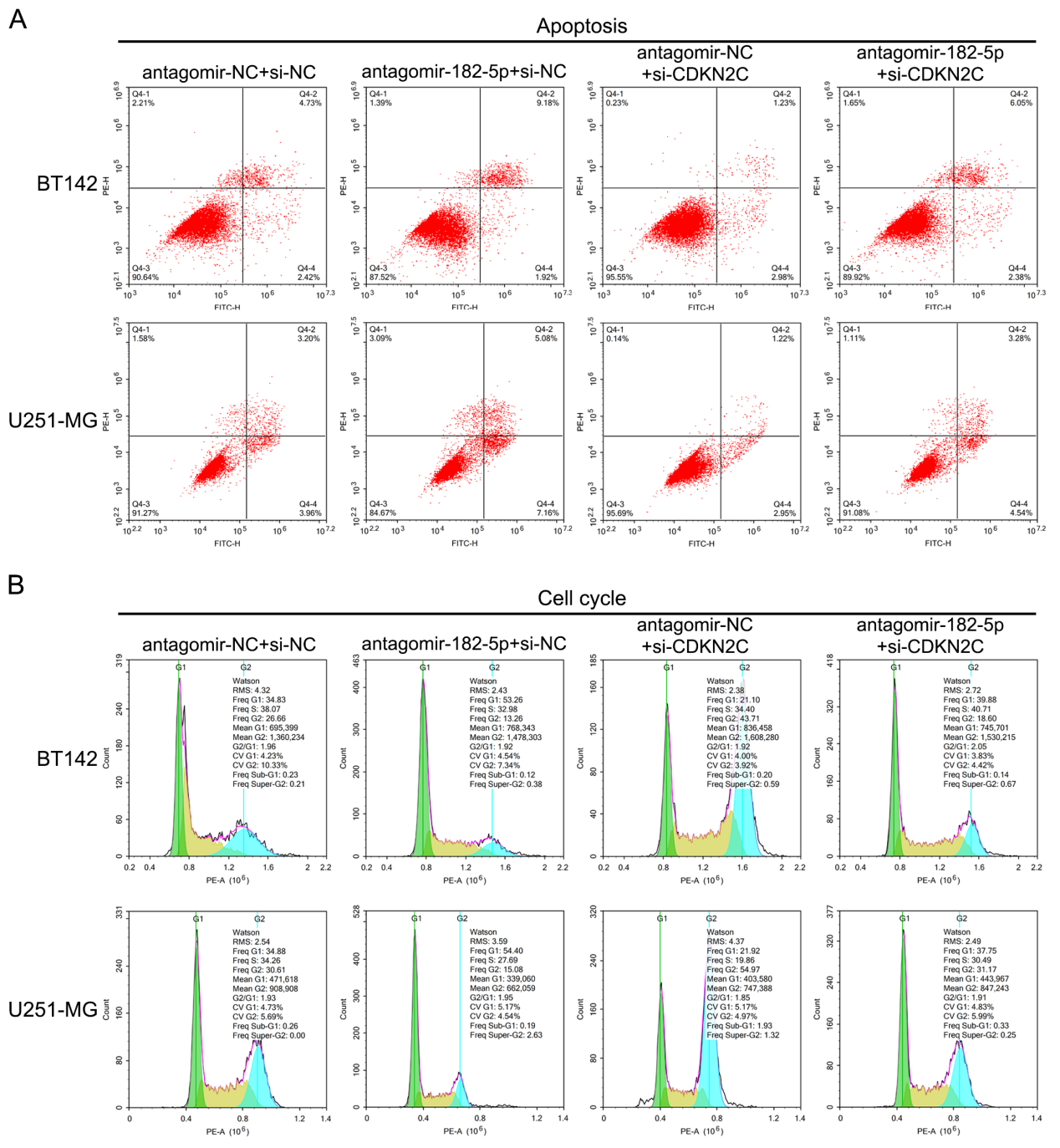


**Fig.S2 Images for cell apoptosis (A) and cell cycle distribution (B) by Flow cytometry in Fig.7C-D.**


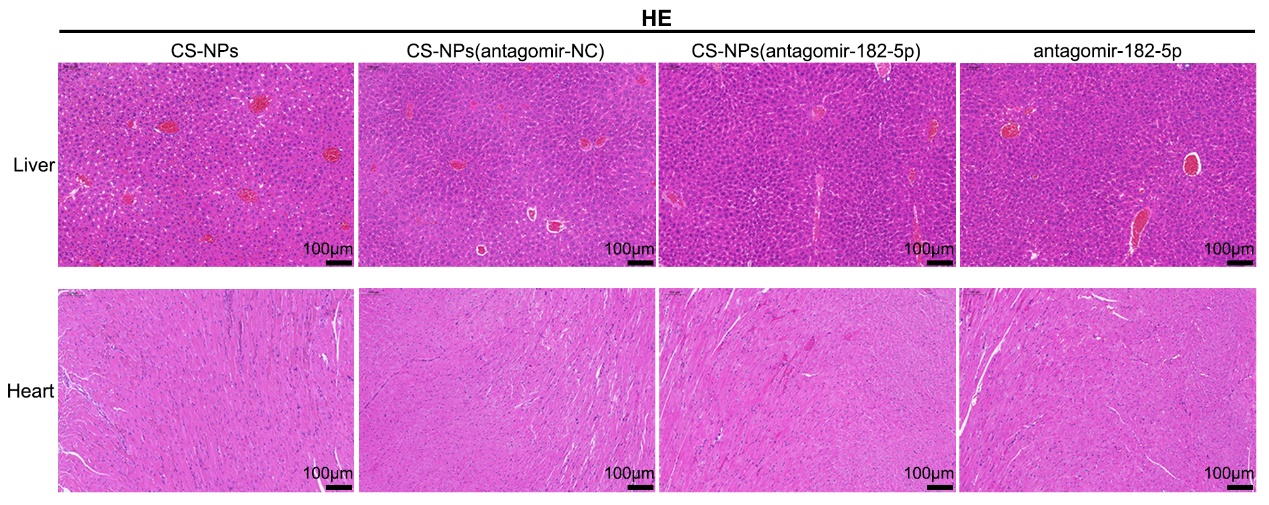


**Fig.S3 Biosafety evaluation** Scale bar = 100 μm.
