# Supplementary material for: IDH1 mutation produces R-2-hydroxyglutarate (R-2HG) and induces mir-182-5p expression to regulate cell cycle and tumor formation in glioma
Source: Biol Res. 2024 May 17;57:30. doi: 10.1186/s40659-024-00512-2 (PMC11100189; doi:10.1186/s40659-024-00512-2)
Supplement: Supplementary file 2 — Supplementary Material 2 [file 40659_2024_512_MOESM2_ESM.docx]

**Table S1. The primer sequences for RT-qPCR and vector sequences in the study**

| **Gene** | **Primer** | **Sequence** **(5'-3')** |
| --- | --- | --- |
| miR-182-5p | Forward | RT-F：  GTCGTATCCAGTGCGTGTCGTGGAGTCGGCAATTGCACTGGATACGACAGTGTG  F：GCTTTGGCAATGGTAGAACT |
|  | Reverse | CAGTGCGTGTCGTGGA |
| pre-miR-182-5p | Forward | GAGCTGCTTGCCTCCCCCCGTTTT |
|  | Reverse | GTGCCGGCTGAGTCCTCGCCCCATA |
| CDKN2C | Forward | ATGATGCGGCCAGAGCAGGTTT |
|  | Reverse | TTCACCAGGAACTCCACCACCC |
| U6 | Forward | CTCGCTTCGGCAGCACA |
|  | Reverse | AACGCTTCACGAATTTGCGT |
| GAPDH | Forward | ACAGCCTCAAGATCATCAGC |
|  | Reverse | GGTCATGAGTCCTTCCACGAT |
| agomir-NC | Forward | UUCUCCGAACGUGUCACGUTT |
|  | Reverse | ACGUGACACGUUCGGAGAATT |
| agomir-182-5p | Forward | UUUGGCAAUGGUAGAACUCACACU |
|  | Reverse | UGUGAGUUCUACCAUUGCCAAAUU |
| antagomir-NC | Forward | CAGUACUUUUGUGUAGUACAA |
|  | Reverse | / |
| antagomir-182-5p | Forward | AGUGUGAGUUCUACCAUUGCCAAA |
|  | Reverse | / |
| si-NC | Forward | UUCUCCGAACGUGUCACGUTT |
|  | Reverse | ACGUGACACGUUCGGAGAATT |
| si-*CDKN2C* | Forward | GGCUUAUGAAUAUAUUUAATT |
|  | Reverse | UUAAAUAUAUUCAUAAGCCTT |
